# Outcomes of Cholelithiasis Among Patients With Sickle Cell Disease: A Tertiary Care Center Experience

**DOI:** 10.7759/cureus.36761

**Published:** 2023-03-27

**Authors:** Bushra Almalki, Sultan Banser, Banan Alghamdi, Majed Almalki, Adel Al-Marzouki, Mohammed Alharthi, Osman Radhwi

**Affiliations:** 1 Hematology, Faculty of Medicine, King Abdulaziz University Hospital, Jeddah, SAU; 2 Hematology Research Unit, King Fahd Center for Medical Research, Jeddah, SAU; 3 Surgery, University of Montreal Health Centre, Montreal, CAN; 4 Digestive Surgery Service, King Abdulaziz University, Jeddah, SAU

**Keywords:** cholecystectomy, sickle cell trait, hemoglobinopathy, sickle cell disease, cholelithiasis

## Abstract

Background: Sickle cell disease (SCD) is a significant health burden in Saudi Arabia that leads to chronic hemolysis with subsequent formation of cholelithiasis. The prevalence of cholelithiasis in the Middle East varies in patients with SCD. The aim of our study was to determine the prevalence of cholelithiasis among SCD patients at a large tertiary care center, King Abdulaziz University Hospital, Jeddah, Saudi Arabia, where more than 300 patients with hemoglobinopathies were followed up.

Methods: In this cross-sectional retrospective study conducted from May 2006 to May 2022, we reviewed 414 patients with SCD who were divided into two groups according to the presence or absence of cholelithiasis. Demographic data, SCD phenotype, splenectomy, cholecystectomy, and hydroxyurea were reviewed from the patient's medical records. They were analyzed to suggest a correlation between the incidence of cholelithiasis and the chances of cholecystectomy.

Results: A total of 414 patients with SCD were reviewed. The mean age of participants was 31 years (10-82), with 52% male. Patients with homozygous sickle hemoglobin (HbSS) constituted 73% of the cohort. The rest (26%) had HbS/β-thalassemia. Thirty-three patients (8%) had splenectomy done. Compliance with hydroxyurea was observed in 174 patients (42%). A total of 64.7% of patients had cholelithiasis (n=269), out of which 159 patients (59.1%) had cholecystectomy done. Surprisingly, a significant association was found between cholecystectomy and the use of hydroxyurea (p=0.003). Additionally, there was a significant association found between the development of cholelithiasis and increasing age (p=0.037).

Conclusion: There was a high prevalence of cholelithiasis found in patients with SCD. It correlated significantly with high-age groups. Further research is warranted to confirm the relationship between hydroxyurea and cholelithiasis.

## Introduction

Sickle cell disease (SCD) is one of the most common inherited hemoglobinopathies worldwide regarding frequency and social impact, recently recognized as a global public health problem by the World Health Organization [1]. It is a monogenic but multisystem disorder with high mortality reaching 100,000 deaths, according to the 2015 Global Burden of Disease report [2]. Hemoglobin S (HbS) mutations in chromosome 11 cause various diseases, including SCD with homozygous sickle hemoglobin (HbSS), HbSC, and HbS beta-thalassemia. When HbS is exposed to deoxygenation, it polymerizes, which is responsible for most of the pathophysiology within red blood cells (RBCs). Therefore, the rigid polymers of HbS disrupt the RBC membrane, shortening the lifespan of RBCs and inducing hemolysis. Furthermore, this makes the RBCs inflexible and abnormally adhesive, which is prone to obstruct blood flow and cause vaso-occlusion that affects all organs because of infarction, repeated ischemia, inflammation, and endothelial dysfunction [2]. SCD is a common hereditary blood disease in the Kingdom of Saudi Arabia (KSA) with a high prevalence in the community, 0.38 per 1000 patients, and it is more common in the Eastern and Southern regions of the country, at a rate of 9.8 per 1000 and 7 per 1000, respectively [3].

Cholelithiasis is one of the common complications of SCD that result either directly from the sickling process or indirectly from chronic hemolysis and multiple blood transfusions. The most common cholelithiasis stone type in SCD is pigmented stone. Its formation is caused by increased bilirubin production due to persistent hemolysis. These pigmented stones can be asymptomatic or have symptoms ranging from biliary colic, cholecystitis, choledocholithiasis to gallstone pancreatitis [4]. Given its potential for severe complications, early diagnosis is essential [4]. Studies have shown that 70% of patients with SCD develop cholelithiasis once in their lifetime, which increases morbidity among these patients [5,6]. A Saudi study included 305 children with SCD aged 1 to 18 years, and cholelithiasis was prevalent in 19.7%. This percentage increased significantly with age ranging from 8.7% in those under 10 to 36% in those aged 15-18 [7].

Most patients are asymptomatic for cholelithiasis; therefore, there is a need for longitudinal follow-up by ultrasonography as a reliable non-invasive method to assess the course of asymptomatic cholelithiasis in addition to other hepatobiliary abnormalities [8]. Regarding symptomatic patients, laparoscopic cholecystectomy is the procedure of choice, but controversy still exists regarding the appropriate treatment for asymptomatic patients with cholelithiasis [9]. Almost half of the asymptomatic patients develop gallbladder complications after three to five years from cholelithiasis [6]. The decision for prophylactic cholecystectomy is debatable. The National Heart, Lung, and Blood Institute of the United States developed evidence-based SCD management guidelines where a watchful wait is strongly recommended [10]. On the other hand, prophylactic laparoscopic cholecystectomy showed shorter hospitalization stays and lower postoperative complication rates compared to symptomatic patients (11.5% vs. 25.5%, respectively) [11].

The aim of this study was to determine the prevalence of cholelithiasis in patients with SCD treated at King Abdulaziz University Hospital (KAUH) in Jeddah, Saudi Arabia, and to explore the possible risk factors for developing such a condition.

## Materials and methods

This cross-sectional, retrospective study was conducted from May 2006 to May 2022 at a tertiary health care center, KAUH, Jeddah, KSA. It involved patients aged 10 years and older with SCD (the required age to be treated by hematologists at KAUH). The medical records, imaging, and hemoglobin electrophoresis workup of 576 patients diagnosed with SCD were reviewed. Patients with sickle cell trait and whose gallbladder status was not determined by abdominal ultrasound (US) or computed tomography (CT) were excluded from our study. Collected data included demographics (age, sex, and SCD type), compliance to hydroxyurea use based on the frequency of outpatient clinic prescription, gall bladder US/CT findings with cholelithiasis or biliary sludge, and surgical history.

Statistical analysis

Data were analyzed using IBM SPSS, version 28.0 (IBM Corp., Armonk, NY). Categorical variables were described using the frequency and percentage. In contrast, continuous quantitative variables were described by the mean and standard deviation (SD) as well as the median and interquartile range (IQR) for abnormally distributed variables (as evidenced by the Shapiro-Wilk test). The Mann-Whitney test was used to compare the mean of a continuous variable between two different groups. The chi-square test was adopted to test for the association between two categorical variables. The Fisher exact test was used to see if there were any associations between the nonrandom categorical variables. Multivariate logistic regression analysis expressed as adjusted odds ratio (AOR) and its 95% confidence interval (CI) was applied to control for the confounding effect. A p-value ≤ 0.05 was considered to indicate statistical significance.

Ethical considerations

The Research Ethics Committee at the Unit of Biomedical Ethics, King Abdulaziz University, Faculty of Medicine, Jeddah, KSA, approved this retrospective study conducted at KAUH, Jeddah (reference number 100-21). The study was conducted in accordance with the guidelines of the Declaration of Helsinki.

## Results

Out of 576 patients, 414 patients with sickle cell anemia/sickle beta-thalassemia were available for analysis. Seventy-seven patients were diagnosed with sickle cell trait, and 85 patients did not have any abdominal US/CT. Their demographic and clinical characteristics are summarized in Table 1. Their age ranged between 10 and 82 years, with an arithmetic mean of 30.81 years and a standard deviation of 9.65 years. More than half (51.4%) of patients were males and Saudi nationals (51.7%). The major blood group was group O (54.4%), followed by group A (30.7%). Patients on hydroxyurea with gallbladder pathology and those who reported a history of splenectomy were 42%, 64.7%, and 8% of patients, respectively. Regarding hemoglobin electrophoresis, most of them belonged to the HbSS type (72.8%).

**Table 1:** Demographic and clinical characteristics of patients with sickle cell anemia/sickle beta-thalassemia (n=414) IQR: inter-quartile range; Hb: hemoglobin *Others: HbSE (n=2), HbSO (n=1), HbSC (n=2), and sickle anemia alpha-thalassemia (n=1)

	Frequency (%)
Gender	
Male	213 (51.4)
Female	201 (48.6)
Age (years)	
Range	10-82
Median (IQR)	31 (23-37.25)
Nationality	
Saudi	214 (51.7)
Non-Saudi	200 (48.3)
Blood group	
A	122 (29.4)
B	42 (10.1)
AB	17 (4.1)
O	217 (52.4)
Missing	16 (4)
Hb electrophoresis	
HbSS	301 (72.8)
HbS/beta-thalassemia	107 (25.8)
Others*	6 (1.4)
On hydroxyurea	
No	240 (58)
Yes	174 (42)
Gallbladder pathology	
No	145 (35.3)
Yes	269 (64.7)
Splenectomy	
No	381 (92)
Yes	33 (8)

History of cholecystectomy

**Figure 1:**
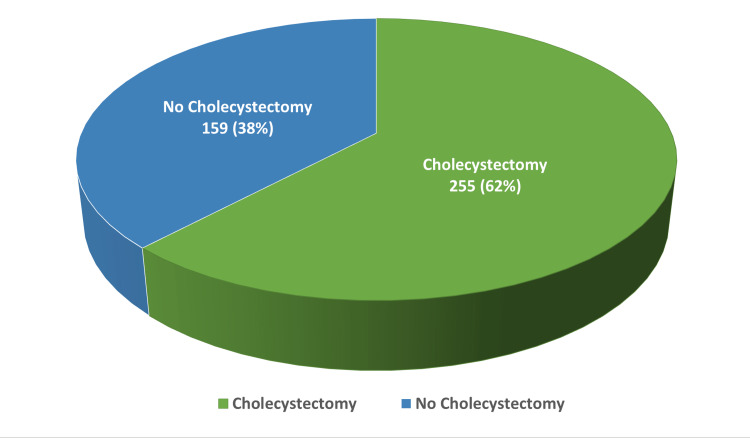
History of cholecystectomy among patients with sickle cell disease

A history of cholecystectomy was reported among more than one-third of patients (38.4%), as illustrated in Figure 1. Patients on hydroxyurea were more likely to have cholecystectomy than their peers (47.1% vs. 32.1%), p=0.002 (Table 2). More than half (59.1%) of patients with gallbladder pathology compared to those without a pathology had a cholecystectomy (p<0.001). Patients with a history of splenectomy were more likely to have cholecystectomy than those without such a history (57.6% vs. 36.7%), p=0.018. Multivariate logistic regression analysis revealed that patients with a history of splenectomy were at an almost threefold risk of having cholecystectomy compared to their counterparts (AOR=2.95; 95% CI: 1.0-8.71, p=0.050) (Table 3). Patients with a history of gallbladder pathology were at a higher risk of cholecystectomy than others (AOR=230.4; 95% CI: 31.52-1684.8, p<0.001). Patients on hydroxyurea were at an almost double risk of having cholecystectomy compared to their peers (AOR=2.19; 95% CI: 1.31-3.64, p=0.003).

**Table 2:** Demographic and clinical factors associated with cholecystectomy among patients with sickle cell anemia/sickle beta-thalassemia IQR: inter-quartile range; Hb: hemoglobin *Others: HbSE (n=2), HbSO (n=1), HbSC (n=2), and sickle anemia alpha-thalassemia (n=1)

	Cholecystectomy		p-value
	No, N=255	Yes, N=159	
Age (years)			
Median (IQR)	31 (23-37)	32 (24-38)	0.241
Gender, N (%)			0.115
Male	139 (65.3)	74 (34.7)	
Female	116 (57.7)	85 (42.3)	
Nationality, N (%)			0.570
Saudi	129 (60.3)	85 (39.7)	
Non-Saudi	126 (63.0)	74 (37.0)	
Blood group, N (%)			0.934
A	73 (59.8)	49 (40.2)	
B	27 (64.3)	15 (35.7)	
AB	11 (64.7)	6 (35.3)	
O	130 (59.9)	87 (40.1)	
Hb electrophoresis, N (%)			0.607
HbSS	181 (60.1)	120 (39.9)	
HbS/beta-thalassemia	70 (65.4)	37 (34.6)	
Others*	4 (66.7)	2 (33.3)	
On hydroxyurea, N (%)			
No	163 (67.9)	77 (32.1)	
Yes	92 (52.9)	82 (47.1)	0.002
Gallbladder pathology, N (%)			
No	145 (100)	0 (0.0)	
Yes	110 (40.9)	159 (59.1)	<0.001
Splenectomy, N (%)			0.018
No	241 (63.3)	140 (36.7)	
Yes	14 (42.4)	19 (57.6)	

**Table 3:** Predictors of cholecystectomy among patients with sickle cell anemia/sickle beta-thalassemia, using multivariate logistic regression analysis AOR: adjusted odds ratio; CI: confidence interval ^a^Reference category

	AOR	95% CI	p-value
Splenectomy			
No^a^	1.0	--	
Yes	2.95	1.00-8.71	0.050
Gallbladder pathology			
No^a^	1.0	--	
Yes	230.4	31.52-1684.8	<0.001
On hydroxyurea			
No^a^	1.0	--	
Yes	2.19	1.31-3.64	0.003

History of cholelithiasis/sludge

**Figure 2:**
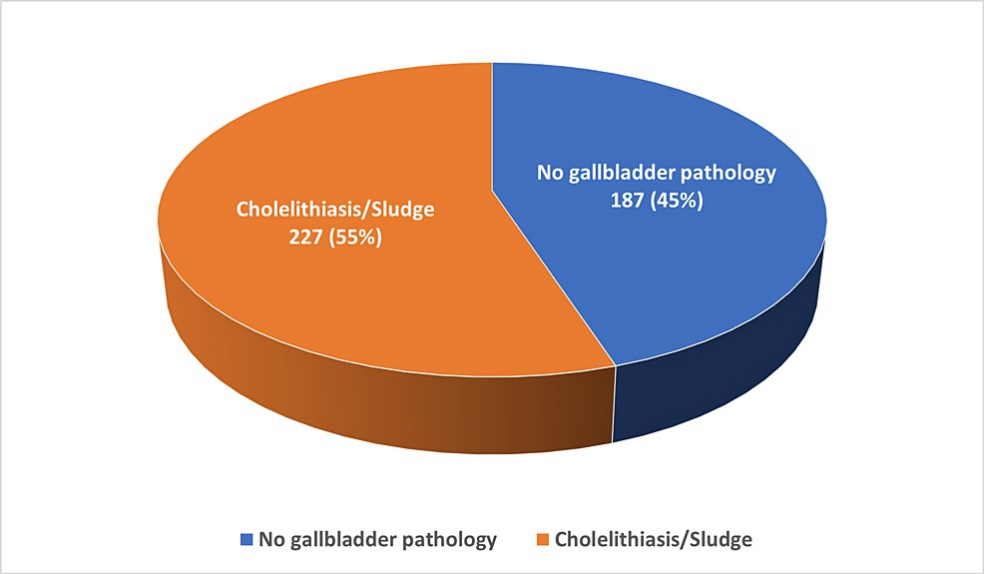
Prevalence of gallbladder pathology among patients with sickle cell disease

As shown in Figure 2, 45.2% of patients had a history of cholelithiasis and/or sludge. The age of patients with a history of cholelithiasis/sludge was significantly lower than that of those without such history; the median (IQR) was 30 (23-36) and 33 (24-39), respectively, p=0.037 (Table 4).

**Table 4:** Demographic and clinical factors associated with cholelithiasis/sludge among patients with sickle cell anemia/sickle beta-thalassemia SD: standard deviation; IQR: inter-quartile range; Hb: hemoglobin *Others: HbSE (n=2), HbSO (n=1), HbSC (n=2), and sickle anemia alpha-thalassemia (n=1)

	Cholelithiasis/sludge		p-value
	No, N=187	Yes, N=227	
Age (years)			
Median (IQRI)	33 (24-39)	30 (23-36)	0.037
Gender, N (%)			0.967
Male	96 (45.1)	117 (54.9)	
Female	91 (45.3)	110 (54.7)	
Nationality, N (%)			0.947
Saudi	97 (45.3)	117 (54.7)	
Non-Saudi	90 (45.0)	110 (55.0)	
Blood group, N (%)			0.663
A	57 (46.7)	65 (53.3)	
B	15 (35.7)	27 (64.3)	
AB	7 (41.2)	10 (58.8)	
O	95 (43.8)	122 (56.2)	
Hb electrophoresis, N (%)			0.225
HbSS	129 (42.9)	172 (57.1)	
HbS/beta-thalassemia	54 (50.5)	53 (49.5)	
Others*	4 (66.7)	2 (33.3)	
On hydroxyurea, N (%)			0.200
No	102 (42.5)	138 (57.5)	
Yes	85 (48.9)	89 (51.1)	
Splenectomy, N (%)			0.690
No	171 (44.9)	210 (55.1)	
Yes	16 (48.5)	17 (51.5)	

## Discussion

According to different epidemiological studies, the prevalence of cholelithiasis in the Middle East varies in patients with SCD, ranging from 11% to 75% [7,8,12-15]. The selection of distinct populations with different age ranges might explain some variations. In our study, the prevalence of cholelithiasis was 54.83%, with a mean age of 30.09±9.40 years. Our study showed that age is a significant risk factor for finding cholelithiasis among patients with SCD. This aligns with the findings by Alhawsawi et al., reporting a significant association between cholelithiasis prevalence and patient age in Saudi patients, with proportions ranging from 11.5% in patients under the age of six to 24.4% in patients aged 6-12 and up to 40.8% in patients over the age of 12 (p=0.018) [13]. Similar findings were found in a research by Attalla, who reported that 0.7% of patients under the age of five had cholelithiasis, 13% of patients between the ages of 5 and 10, and 33% between the ages of 10 and 16 [14].

In the general population, females have a two- to threefold increase in the risk of developing cholelithiasis [16]. In our cohort of patients, no significant association was found in the prevalence of cholelithiasis between males and females (51.54% and 48.46%, respectively), and similar results were observed in other studies [12,15].

Although patients with beta-thalassemia are known to be in a chronic hemolysis state, our patients with HbSS were reported to have a higher prevalence of cholelithiasis than participants with HbS beta-thalassemia. However, these differences were not statistically significant (p>0.05). Similar findings were reported by Alhawsawi et al. [13].

We found a non-significant difference in the prevalence of cholelithiasis between patients who used hydroxyurea (51.1%) and those who did not (57.5%). This goes along with the findings of Alhawsawi et al. and Martins et al. who reported no association between consuming hydroxyurea and developing cholelithiasis [13,17]. Multiple variables can play into this finding. These include, but are not limited to, the constant use of hydroxyurea with the maximum tolerated doses, the genetic predisposition to cholelithiasis, and the presence of an undiagnosed form of hemolytic anemia.

The decision for cholecystectomy varied depending on the patient's presentation and the surgical opinion at the time. Hence, capturing the reason for cholecystectomy was difficult. Notably, the use of hydroxyurea was found to be associated with the cohort's rate for cholecystectomy (p=0.003). This statistically significant finding can be partially explained by the fact that the more severe the clinical course of the disease, the more the patient will be on hydroxyurea.

Study limitations

We must address some limitations in this study. First, its retrospective nature led to the exclusion of many patients with missing data. Second, the reliance on the use of hydroxyurea subjectively and whether patients had cholelithiasis before taking hydroxyurea can bias our conclusions. Third, it was difficult to capture how many units of blood were transfused annually as our patients can access medical care in areas outside our facility.

## Conclusions

In the present study, our results showed the prevalence of cholelithiasis (31.4%) in patients with SCD, with the incidence increasing with age, typically rising after the age of 30 years. Abdominal ultrasonography investigations and routine clinical follow-ups are highly advised to aid in the early detection of cholelithiasis and the prevention of associated consequences. It is recommended to perform more research to determine the appropriate age for screening and the timing for prophylactic cholecystectomy, and assess the association between hydroxyurea and cholelithiasis.
